# Effect of Serum Ferritin on the Association Between Coffee Intake and Hyperuricemia Among American Women: The National Health and Nutrition Examination Survey

**DOI:** 10.7759/cureus.13855

**Published:** 2021-03-12

**Authors:** Dayawa D Agoons, Batakeh B. Agoons, Arnaud D. Kaze, Saketram Komanduri

**Affiliations:** 1 Internal Medicine, University of Pittsburgh Medical Center Pinnacle Hospital, Harrisburg, USA; 2 Department of Internal Medicine, Faculty of Medicine and Biomedical Sciences, University of Yaounde, Yaoundé, CMR; 3 Internal Medicine, Johns Hopkins School of Medicine, Baltimore, USA; 4 Medicine, Sovah Health, Virginia, USA

**Keywords:** gout, arthritis, crystal arthropathies

## Abstract

Background

Accruing evidence suggests an inverse relationship between coffee intake and serum uric acid. The mechanism(s) explaining this inverse relationship remains elusive. The aim of this study was to assess if the association between coffee intake and hyperuricemia is mediated via serum ferritin in women.

Methods

We pooled data from the 2003 to 2006 National Health and Nutrition Examination Survey (NHANES). We included women with complete information on all key variables. Coffee intake was classified as none, <1 cup/day, 1-3 cups/day, and ≥4 cups/day. Hyperuricemia was defined as serum uric acid >5.7 mg/dL. We assessed the association between coffee intake and hyperuricemia using logistic regression. Path analysis was used to examine whether serum ferritin mediated the effect of coffee on hyperuricemia.

Results

Among 2,139 women (mean age: 31.2 years [SD: 9.2]), mean serum uric acid was 4.4 mg/dL (SD: 1.0), and 227 (10.6%) had hyperuricemia. In multivariate logistic regression models, intake of ≥4 cups/day of coffee was associated with lower odds of hyperuricemia (OR 0.28 [95% CI: 0.09, 091], P=0.035). The total direct and indirect effect of coffee on hyperuricemia via serum ferritin was −0.16, P=0.009 and −8.1 × 10^−^^3^, P=0.204, respectively.

Conclusion

Among women, moderate coffee consumption was inversely related to hyperuricemia by direct effect, rather than indirectly through the effects of serum ferritin. These findings suggest that serum ferritin does not mediate the inverse association between coffee and hyperuricemia in women.

## Introduction

Coffee is a commonly consumed beverage around the world. An estimated one in two adults in the United States (US) drinks coffee, with an average per capita intake of two cups a day [1,2]. Accruing evidence from traditional epidemiological studies has suggested a protective effect of coffee consumption on serum uric acid and gout [3-5]. Indeed, there are also Mendelian randomization studies that have suggested a causal association between coffee consumption and gout [6]. However, the mechanism(s) explaining this inverse relationship remain elusive.

There is evidence showing that coffee consumption interferes with iron absorption in the gut [7,8]. Morck et al. reported up to a 39% decrease in iron absorption following coffee intake [9]. Interestingly, in a recent study among Korean adults, coffee consumption was associated with lower serum ferritin levels, an index of iron stores in the body [10]. In other studies, serum ferritin has been positively correlated with serum uric acid levels, with increased ferritin levels associated with gout and frequency of gout flare [11,12]. Whether serum ferritin mediates the effect of coffee consumption on uric acid and gout is unclear.

Using data from the National Health and Nutrition Examination Survey (NHANES), we sought to determine if the inverse association between coffee consumption and uric acid is mediated through serum ferritin among women. We hypothesized that coffee consumption would be associated with lower levels of serum ferritin which would, in turn, be associated with a lower risk of hyperuricemia.

## Materials and methods

Study design and population

We pooled data from 2003 to 2004 and 2005 to 2006 NHANES. The NHANES is a large, nationally representative survey of the civilian non-institutionalized adult population in the United States conducted by the Centers for Disease Control and Protection [13]. The survey consists of demographic, dietary, laboratory, and questionnaire data collected by trained personnel. Serum ferritin measurements were not systematically available for men. We, therefore, limited our analyses to adult women aged 18 years and over. Of the 11,183 adults in the NHANES 2003-2004 and 2005-2006, we excluded 9,044 with missing data, leaving a total of 2,139 study participants in the final analysis.

Assessment of coffee consumption

Coffee consumption was determined from participants’ responses to a food frequency questionnaire (FFQ) which collected information on their frequency of food consumption over the past 12 months. In the NHANES FFQ, there are 10 coffee consumption frequency categories including none, <1 cup/month, 1-3 cups/month, 1 cup/week, 2-4 cups/week, 5-6 cups/week, 1 cup/day, 2-3 cups/day, 4-5 cups/day, and ≥6 cups/day [13]. In our analyses, we classified coffee intake into four categories: none, less than 1 cup/day, 1-3 cups/day, and ≥ 4 cups/day.

Assessment of serum uric acid

Serum uric acid was measured by oxidation technique using the enzyme uricase to produce allantoin and hydrogen peroxide. In a reaction catalyzed by peroxidase, the hydrogen peroxide forms a colored product whose absorbance is proportional to the concentration of uric acid in the serum sample [13]. Uric acid was measured in milligrams per deciliter (mg/dL). Hyperuricemia was defined as serum uric acid >5.7 mg/dL [14].

Assessment of serum ferritin

Serum samples were maintained frozen at −20 °C until analysis. Serum ferritin was measured by immune-turbidimetry using the Roche/Hitachi 912 clinical analyzer. Ferritin antigen-antibody complexes formed are proportional to the concentration of ferritin in the serum sample [13]. Ferritin was measured in nanograms per milliliter (ng/mL).

Assessment of covariates

Data on past medical history, smoking status, alcohol consumption, and level of education, were obtained in interviews using standardized questionnaires. Body mass index (BMI) was computed as weight in kilograms divided by the square of height in meters. Waist circumference was the average of two readings measured around the umbilicus and in the upright position and measured to the nearest 1 cm. Education level was categorized as having less than high school education, high school graduate education, or some college education or higher. Participants who responded “yes” to the question “Do you now smoke cigarettes?” were coded as current smokers. Alcohol consumption was defined as ≥12 alcohol drinks per year. Glomerular filtration rate (GFR) was estimated using the Chronic Kidney Disease Epidemiology Collaboration (CKD-EPI) equation: GFR = 141 * min(Scr/κ,1)α * max(Scr/κ, 1)-1.209 * 0.993Age * 1.018 [if female] * 1.159 [if black] [15].

Statistical analyses

Coffee consumption was categorized into four groups: none, <1 cup/day, 1-3 cups/day, and ≥4 cups/day. Serum uric acid was analyzed both as a continuous and as a categorical variable (hyperuricemia: uric acid >5.7 mg/dL). Baseline characteristics were presented as mean (SD) and median (IQR) for continuous variables as appropriate, and percentage for categorical variables. Participant characteristics were compared using the analysis of variance (ANOVA), Kruskal-Wallis test, and χ^2^ test as appropriate.

**Figure 1:**
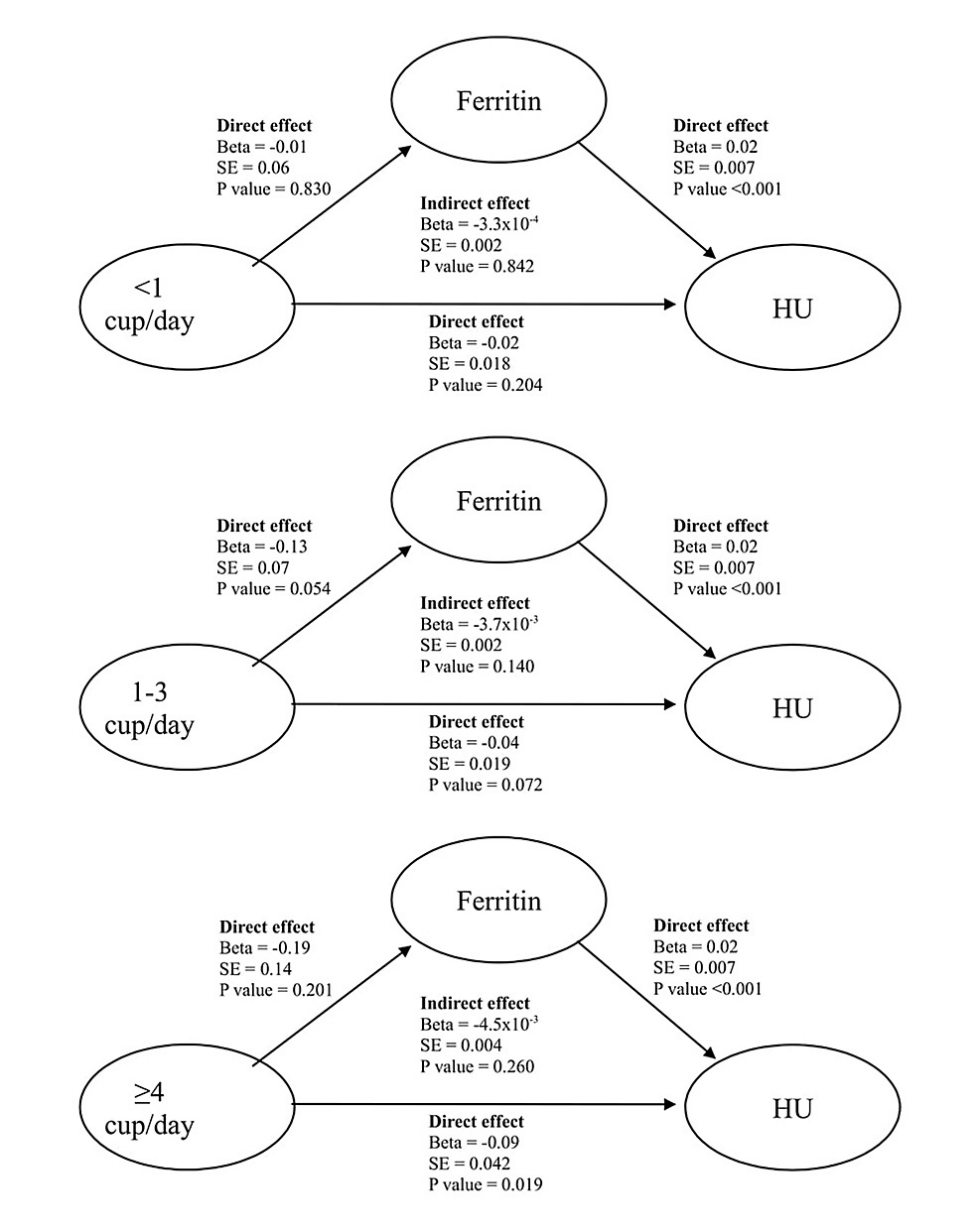
Summary of mediation analysis Standardized pathway coefficients are shown by category of coffee consumption. Effect sizes are shown adjusted for age, alcohol consumption, smoking, diabetes, hypertension, and eGFR. SE: standard error; HU: hyperuricemia.

The relation between coffee consumption and serum uric acid and hyperuricemia was assessed with linear and logistic regression, respectively. The relation between coffee consumption and serum ferritin, and between serum ferritin and hyperuricemia was assessed using linear regression. We conducted sequential adjustments, initially adjusting for age (model 1). We also evaluated the following additional models: model 1 with additional adjustment for alcohol consumption, smoking, GFR, history of diabetes mellitus and hypertension (model 2), and model 2 with additional adjustment for BMI (model 3). Path analysis with 2000 bootstraps was used to examine whether the effect of coffee intake on hyperuricemia was mediated through serum ferritin (Figure 1). A two-sided p-value <0.05 was considered statistically significant. All statistical analyses were performed using Stata version 15 (StataCorp, College Station, TX).

## Results

Characteristics of study participants

Table 1 summarizes the baseline characteristics of participants by categories of coffee intake. The mean age of the participants was 31.2 (SD: 9.6) years. The mean serum uric level was 4.38 mg/dL. Participants in the highest coffee intake category were older, more likely to be current smokers, alcohol consumers, have a lower estimated glomerular filtration rate (eGFR), and have high serum ferritin levels. Table 1 shows the baseline characteristics of the participants by hyperuricemia. Of the 2,139 participants, 227 (10.6%) had hyperuricemia. Participants with hyperuricemia were older, had a higher BMI, lower eGFR, higher serum ferritin level, and reported hypertension and diabetes more compared to those without hyperuricemia.

**Table 1:** Baseline characteristics of study participants by coffee intake, number of cups/day Data are mean (SD), median (IQR), or proportion (%) as appropriate. SD: standard deviation; IQR: interquartile range; LDL-C: low-density lipoprotein cholesterol; eGFR: estimated glomerular filtration rate. *Alcohol consumption was defined as ≥12 alcoholic drinks/year. ^§^GFR was estimated using the Chronic Kidney Disease Epidemiology Collaboration (CKD-EPI).

	None	<1 cup/day	1-3 cups/day	≥4 cups/day	P-value
Characteristics	(n = 819)	(n = 750)	(n = 504)	(n = 66)	<0.001
Age (years)	28.8 (9.2)	29.8 (9.2)	36.1 (8.7)	39.4 (7.0)	0.683
Education (%)					
Less than high school	19.1	23.1	20.7	21.5	
High school graduate	22.7	19.3	21.1	24.6	
Attended a college or higher	58.2	57.5	58.3	53.9	
Anthropometric variables					
Body mass index (kg/m^2^)	28.9 (7.8)	28.4 (7.5)	27.8 (6.5)	28.1 (6.9)	0.116
Waist circumference (cm)	95.0 (17.7)	93.6 (16.6)	92.4 (14.8)	92.4 (15.4)	0.036
Lifestyle variables					
Current smoker (%)	54.2	54.4	57.9	80.4	0.006
Alcohol consumption* (%)	52.2	63.5	72.9	71.4	<0.001
Hemodynamic variables					
Systolic blood pressure (mmHg)	111.6 (13.4)	111.2 (14.0)	114.1 (15.9)	112.1 (13.9)	0.087
Diastolic blood pressure (mmHg)	66.6 (12.5)	65.4 (12.4)	69.1 (11.5)	69.9 (8.6)	0.002
Hypertension, %	14.2	10.8	14.8	12.1	0.063
Metabolic variables					
Total cholesterol (mg/dL)	193.9 (44.1)	196.0 (48.5)	195.8 (41.2)	192.8 (36.4)	0.903
LDL-C (mg/dL)	110.4 (31.4)	103.5 (31.3)	111.3 (31.8)	129.2 (32.7)	0.003
Diabetes mellitus (%)	3.4	2.7	3.2	6.1	0.718
eGFR, ml/min/1.73 m^2§^	116.1 (20.1)	114.7 (17.9)	105.7 (17.8)	102.5 (16.5)	<0.001
Ferritin (ng/dL)	33 (17-64)	35 (18-63)	44 (23-71)	42 (23-77)	0.393
Uric acid (mg/dL)	4.4 (1.1)	4.4 (1.0)	4.4 (1.0)	4.3 (0.9)	0.766

Association of coffee intake with serum uric acid

There was no significant association between coffee intake and serum uric acid (Table 2). After adjusting for age, intake of ≥4 cups/day of coffee was associated with a decrease in the odds of hyperuricemia (OR 0.28 [95% CI: 0.09, 0.91], P=0.035). Additional adjustments for alcohol consumption, smoking, diabetes, hypertension, and eGFR did not change the magnitude and significance of this result. This inverse relationship was not significant for coffee consumption of <1 or 1-3 cups/day after adjusting for potential confounders (Table 3).

**Table 2:** Association of coffee intake with serum uric acid, mg/dL Model 1: adjusted for age; model 2: model 1 + adjusted for alcohol consumption, smoking, diabetes, hypertension, and eGFR; model 3: model 2 + adjusted for BMI.

Coffee intake	Model 1		Model 2		Model 3	
	β Coef. (95% CI)	P-value	β Coef. (95% CI)	P-value	β Coef. (95% CI)	P-value
None	Ref	…	Ref	…	Ref	…
<1 cup/day	−0.04 (−0.15, 0.06)	0.429	−0.03 (−0.15, 0.09)	0.609	−0.01 (−0.11, 0.09)	0.518
1-3 cups/day	−0.05 (−0.17, 0.07)	0.386	−0.07 (−0.20, 0.06)	0.321	0.04 (−0.08, 0.16)	0.497
≥4 cups/day	−0.19 (−0.46, 0.07)	0.156	−0.26 (−0.53, 0.01)	0.063	−0.15 (−0.45, 0.09)	0.228
P-value of trend		0.175		0.108		0.984

**Table 3:** Association of coffee intake with hyperuricemia, mg/dL Model 1: adjusted for age; model 2: model 1 + adjusted for alcohol consumption, smoking, diabetes, hypertension, and eGFR; model 3: model 2 + adjusted for BMI.

Coffee intake	Model 1		Model 2		Model 3	
	OR (95% CI)	P-value	OR (95% CI)	P-value	OR (95% CI)	P-value
None	Ref	…	Ref	…	Ref	…
<1 cup/day	0.79 (0.58, 1.09)	0.150	0.83 (0.57, 1.22)	0.338	0.83 (0.56, 1.25)	0.337
1-3 cups/day	0.69 (0.47, 1.01)	0.051	0.69 (0.46, 1.06)	0.091	0.86 (0.55, 1.34)	0.15
≥4 cups/day	0.28 (0.09, 0.91)	0.035	0.28 (0.08, 0.95)	0.042	0.32 (0.09, 1.14)	0.079
P-value of trend		0.008		0.020		0.173

Association of serum ferritin with serum uric acid

Table 4 shows the association of serum ferritin with serum uric acid and hyperuricemia. In age-adjusted models, 1 SD increase in serum ferritin was associated with 0.16 mg/dL increase in serum uric acid (β Coef. 0.16 [95% CI: 0.12, 0.20], P<0.001). Further adjustment for other confounders did not change the magnitude and significance of these findings (Table 4). Similar results were observed between serum ferritin and hyperuricemia. After adjustment for age, alcohol consumption, smoking, diabetes, hypertension, and eGFR (Table 4, model 2), 1 SD increase in serum ferritin was associated with a 21% increase in the odds of hyperuricemia (OR 1.21 [95% CI: 1.08, 1.36], P = 0.001). In multivariate regression models adjusting for potential confounders, coffee consumption was not associated with serum ferritin levels (Table 5).

**Table 4:** Association of serum ferritin (per SD increment) with serum uric acid and hyperuricemia Model 1: adjusted for age; model 2: model 1 + adjusted for alcohol consumption, smoking, diabetes, hypertension, and eGFR; model 3: model 2 + adjusted for BMI.

	Uric acid		Hyperuricemia	
	β Coef. (95% CI)	P-value	OR (95% CI)	P-value
Model 1	0.16 (0.12, 0.20)	<0.001	1.25 (1.12, 1.39)	<0.001
Model 2	0.13 (0.09, 0.18)	<0.001	1.21 (1.08, 1.36)	0.001
Model 3	0.10 (0.06, 0.14)	<0.001	1.15 (1.01, 1.30)	0.032

**Table 5:** Association of coffee intake with the serum ferritin, ng/mL Model 1: adjusted for age; model 2: model 1 + adjusted for alcohol consumption, smoking, diabetes, hypertension, and eGFR; model 3: model 2 + adjusted for BMI.

Coffee intake	Model 1		Model 2		Model 3	
	β Coef. (95% CI)	P-value	β Coef. (95% CI)	P-value	β Coef. (95% CI)	P-value
None	Ref	…	Ref	…	Ref	…
<1 cup/day	−1.92 (−7.69, 3.83)	0.511	−0.79 (−8.09, 6.50)	0.831	−0.31 (−7.61, 6.99)	0.935
1-3 cups/day	−6.05 (−12.7, 0.69)	0.078	−7.97 (−16.1, 0.17)	0.055	−6.18 (−14.4, 1.99)	0.139
≥4 cups/day	−9.11 (−23.9, 5.73)	0.229	−11.0 (−27.9, 5.90)	0.201	−10.5 (−27.6, 6.51)	0.226
P-value of trend		0.055		0.042		0.097

The results of mediation analyses are illustrated in Figure 1. There was a significant total direct effect of coffee consumption on hyperuricemia (β -0.16, standard error [SE] 0.06, P<0.01). A significant direct effect of ≥4 cups/day of coffee on hyperuricemia was also demonstrated (β −0.09, SE 0.04, P = 0.02). The direct effect of <1 cup/day or 1-3 cups/day of coffee intake on hyperuricemia was not significant (β −0.02, SE 0.01, P = 0.204, and β -0.04, SE 0.02, P = 0.072, respectively). The total and individual indirect effect of all three categories of coffee intake on hyperuricemia through serum ferritin was not statistically significant (all P>0.05). Only 4.9% of the total effect (total indirect effect/sum of total indirect and direct effect) of coffee consumption on hyperuricemia was mediated via serum ferritin.

## Discussion

In this cross-sectional study of a group of healthy US women, we sought to assess if serum ferritin mediates the inverse relationship between coffee consumption and hyperuricemia. This study provides further evidence that coffee intake is inversely related to hyperuricemia. We found that consumption of ≥4 cups/day of coffee was associated with reduced odds of hyperuricemia. There was a significant total direct effect of coffee consumption on hyperuricemia, especially of ≥4 cups/day, while the indirect effect of coffee intake on hyperuricemia through serum ferritin was not significant. Our findings suggest that the putative protective effect of coffee intake on hyperuricemia is not mediated through serum ferritin in women.

Our findings are similar to those of previous studies that have examined the relationship between coffee and uric acid/hyperuricemia and also between ferritin and uric acid [11,12,16,17]. Studies evaluating the association between coffee intake and markers of iron metabolism have been conflicting. Fleming et al. [18] and Sung et al. [10] found an inverse association between coffee intake and serum ferritin. However, Delley et al. [7] did not find any significant difference in plasma ferritin between the intervention and control group in a randomized clinical trial that sought to assess the effect of discontinuing coffee on iron status among toddlers. Similar to our findings, previous studies have also shown a significant association between serum ferritin and uric acid and hyperuricemia [19,20].

The mechanism(s) explaining the association between coffee intake and serum uric and gout remains poorly understood. Some animal and human studies have suggested as a plausible mechanism that coffee may confer its protective effect on serum uric acid by reducing glucose, insulin concentration, and insulin resistance [21-23]. Caffeine, a major ingredient of coffee belongs to the family of methylxanthines and is a competitive inhibitor of xanthine oxidase, an enzyme responsible for the production of uric acid [24]. A modest significant association was shown between decaffeinated coffee and uric acid [25] suggesting that components of coffee other than caffeine may also contribute to the putative protective effect of coffee on uric acid. To the best of our knowledge, this is the first study to assess the effect of serum ferritin as a potential mediator of the inverse relationship between coffee consumption and hyperuricemia. Coffee is rich in chlorogenic acid, a phenolic compound and potent antioxidant [1]. Phenols are a potent inhibitor of non-heme iron absorption and have been shown to reduce iron absorption from a test meal by up to 60% [26]. This study was carried out on the assumption that coffee consumption decreases iron absorption, and thus ferritin stores, which in turn decreases serum uric acid levels. However, our results show a non-significant indirect effect of each of the three coffee consumption categories (<1, 1-3, and ≥4 cups/day) on hyperuricemia through serum ferritin. Interestingly, we found that only 4.9% of the total effect of coffee intake on hyperuricemia was mediated via serum ferritin. These findings suggest that serum ferritin does not seem to be involved in this suggested beneficial effect.

Our findings provide data to inform future research aimed at exploring the potential mechanism(s) of the putative protective effect of coffee intake on serum uric acid and gout. Serum ferritin levels could be significantly different between men, pre-menopausal and post-menopausal women. In one study, pre-menopausal women had the lowest serum ferritin level compared to post-menopausal women and men [27]. Hence, it is plausible that the coffee - uric acid/gout relationship might differ depending on differing ferritin levels in different populations. Therefore, it is important to confirm our findings with studies in other populations such as men and post-menopausal women.

Our study has several limitations. First, our study population was limited to women aged 18 to 49 years. Thus, our results may not be generalizable to other populations such as men and post-menopausal women. Second, this was a cross-sectional study; hence, it is susceptible to the problems of residual confounding and reverse causation and therefore cannot establish causality. Third, coffee intake was ascertained using an FFQ and may be subject to recall bias and misclassification. Fourth, data on uric acid lowering medications such as allopurinol was not systematically available in our study population, hence precluding our ability to account for this potential confounder in our analyses.

Despite these limitations, our study has multiple strengths. First, this study was performed in a large and diverse nationally representative sample of adult women in the United States. Second, our study population was composed of healthy adult women free from factors such as ongoing infection, inflammation, and liver disease which could affect the accuracy of serum ferritin level measurement. Third, coffee intake (exposure variable) was ascertained using standardized FFQ. Although the potential for recall bias exists, it is less likely that serum uric acid and ferritin level measurements were systematically impacted by this given that these measurements were performed after the household interviews in the NHANES.

## Conclusions

In summary, we found that moderate coffee intake was associated with reduced odds of hyperuricemia in women. The indirect effect of coffee on hyperuricemia via serum ferritin was not significant. Only 4.9% of the total effect of coffee on hyperuricemia was mediated through serum ferritin. Our results suggest that the putative protective effect of coffee on hyperuricemia is not mediated through serum ferritin in women. Further studies are needed to clarify the mechanisms underlying the inverse association between coffee and uric acid.
